# A Multiple Identity Approach to Gender: Identification with Women, Identification with Feminists, and Their Interaction

**DOI:** 10.3389/fpsyg.2017.01019

**Published:** 2017-06-30

**Authors:** Jolien A. van Breen, Russell Spears, Toon Kuppens, Soledad de Lemus

**Affiliations:** ^1^Department of Social Psychology, University of GroningenGroningen, Netherlands; ^2^Departamento de Psicología Social, Universidad de GranadaGranada, Spain

**Keywords:** gender, multiple identities, social identity, group membership, identification with women, identification with feminists, femininity, stereotypes

## Abstract

Across four studies, we examine multiple identities in the context of gender and propose that women's attitudes toward gender group membership are governed by two largely orthogonal dimensions of gender identity: identification with women and identification with feminists. We argue that identification with women reflects attitudes toward the *content* society gives to group membership: what does it mean to be a woman in terms of group characteristics, interests and values? Identification with feminists, on the other hand, is a politicized identity dimension reflecting attitudes toward the social position of the group: what does it mean to be a woman in terms of disadvantage, inequality, and relative status? We examine the utility of this multiple identity approach in four studies. Study 1 showed that identification with women reflects attitudes toward group characteristics, such as femininity and self-stereotyping, while identification with feminists reflects attitudes toward the group's social position, such as perceived sexism. The two dimensions are shown to be largely independent, and as such provide support for the multiple identity approach. In Studies 2–4, we examine the utility of this multiple identity approach in predicting qualitative differences in gender attitudes. Results show that specific combinations of identification with women and feminists predicted attitudes toward collective action and gender stereotypes. Higher identification with feminists led to endorsement of radical collective action (Study 2) and critical attitudes toward gender stereotypes (Studies 3–4), especially at lower levels of identification with women. The different combinations of high vs. low identification with women and feminists can be thought of as reflecting four theoretical identity “types.” A woman can be (1) strongly identified with neither women nor feminists (“low identifier”), (2) strongly identified with women but less so with feminists (“traditional identifier”), (3) strongly identified with both women *and* feminists (“dual identifier”), or (4) strongly identified with feminists but less so with women (“distinctive feminist”). In sum, by considering identification with women and identification with feminists as multiple identities we aim to show how the multiple identity approach predicts distinct attitudes to gender issues and offer a new perspective on gender identity.

## Introduction

Since the 1980s there has been increasing attention to the complexities of gender identity, acknowledging that, like many other social identities, gender has a strong cultural component, and is not a straightforward biological fact (Unger, 1979; Marecek et al., 2004). Here we examine women's attitudes toward gender group membership, and argue that these attitudes are governed by multiple identities: identification with women and identification with feminists. We contrast this multiple identity approach with other notable multicomponent approaches to gender identity and argue that the multiple identity approach is simple, while allowing for some new nuances in gender identity compared to previous models. Importantly, this approach helps us understand why being feminine and feminist are not mutually exclusive.

We do not consider here the personal, social and biological factors that determine an individual's gender identity, but rather study women's attitudes toward the socially shared aspect of gender group membership. What does it mean to be a member of the social category of women? An important aspect of the reasoning we present here is that an individual is not entirely free to construct the meaning of group membership as they please. Instead, the meaning of group membership is constructed at the societal level and to a large extent socially shared (Moscovici, 1988; Crocker, 1999). We are interested in how people respond to the social construction of a group to which they belong. We believe that considering identification with women and identification with feminists as separable components of gender identity can offer interesting new perspectives on attitudes toward gender group membership.

The idea that gender identity is multidimensional is reflected in many different models (Condor, 1986; Cameron and Lalonde, 2001; Egan and Perry, 2001; Becker and Wagner, 2009), and an important question arising from such approaches is how the dimensions combine and interact. Many models (Condor, 1986; Henderson-King and Stewart, 1994; Cameron and Lalonde, 2001) discuss evidence that high identification with women can be combined with different gender ideologies (e.g., traditional, progressive, feminist). However, if the gender dimensions are seen as independent, then this means that it should also be possible for the same (feminist) ideology to be combined with both high and low identification with women. Yet, few models discuss this option. One influential model that *has* explicitly conceptualized gender identity as composed of two independent dimensions is the Gender Identity Model (GIM, Becker and Wagner, 2009). The GIM aims to explain endorsement of sexism and support for collective action, and distinguishes between (1) identity *content*, a preference for traditional vs. progressive gender roles, and (2) identity *strength*, measured as identification with women. That is, though the GIM postulates two independent dimensions, only one of these dimensions is a content dimension (traditional vs. progressive), while the other, identification with women, reflects identity *strength*. In the current studies we propose that identification with women not only reflects identity strength but also has implications for the content of gender identity. That is, our approach incorporates content for both dimensions.

Specifically, we suggest that the content associated with identification with women centers on group characteristics and attributes: what does it mean to be a woman in terms of one's characteristics, traits, interests and values? For instance, key group attributes may include being warm and caring (Fiske et al., 2002; Chen et al., 2004). Although identity content is likely to be socially shared to some degree, individuals can differ in the extent to which they accept or internalize society's view of the group, which is reflected in their degree of identification (Ellemers et al., 2002). There is evidence that those who identify strongly with their group are more likely to self-stereotype, and consider themselves more typical of the group (Turner et al., 1987; Spears et al., 1997, 2001; Leach et al., 2008). Chen et al. (2004) showed that, when asked to list 5 traits that are most typical of women as a group, those who were strongly identified with women listed the same traits as those who were less identified with women, providing evidence that this perception was socially shared. However, those who were strongly identified with women were more likely to say that (positive) traits that defined the group also defined *themselves* (Chen et al., 2004), than those were less committed to women as a group. Based on these previous findings, we suggest that the content associated with identification with women is socially constructed around *group characteristics*. Those who are highly identified with women place high importance on traits and characteristics that society considers gender-typical, which we expect to translate to increased tendencies to self-stereotype, and increased perceptions of femininity, compared to those who are less strongly identified with women.

Alongside the characteristics associated with the group, the meaning of group membership also includes the place of the group within the larger social system (Livingstone et al., 2009). What does it mean to be a woman in terms of relative status, social (in) equality, and disadvantage? We argue that attitudes toward such (politicized) identity content are reflected in identification with feminists. In line with this notion, previous research has shown that identification with feminists is related to increased perceptions of sexism in society (Henderson-King and Stewart, 1994), discontent with current power distributions and the status quo (Reid and Purcell, 2004), and increased involvement in collective action (Liss et al., 2004; Nelson et al., 2008; Yoder et al., 2011). Based on these previous findings, we argue that the content of feminist identification is socially constructed around disapproval of the disadvantaged social position of women as a group. An individual's degree of identification with feminists reflects the importance they place on these issues. Those who are strongly identified with feminists have internalized the values of feminism, reject the gender status quo, and consider women to be disadvantaged in comparison to men.

In sum, we propose that identification with women and identification with feminists reflect attitudes toward different components of the social construction of gender. If we think of identification with women as relating to *what the group is*, then we can think of identification with feminists as relating to *how the group is doing* in relation to other groups. The level of identification with each of these identities reflects the extent to which a person has accepted and internalized the content associated with that identity. In line with the notion that identification with women and identification with feminists are separable components of gender identity, previous research has found that the correlation between them is very small (Roy et al., 2007).

One benefit of a model in which identification with women and identification with feminists are largely independent, but also associated with specific identity content, is that different combinations of the identities allow for additional nuances in gender identity content. For instance, this perspective allows for high identification with women, without assuming that this will necessarily lead to politicization. Relatedly, those who identify strongly with feminists may differ in their identification with women, which we expect to translate (*inter alia*) to differences in the importance they place on “femininity.” Thus, our multiple identities approach explicitly allows for the possibility that femininity (related to identification with women) could co-exist with feminist identification. Such a distinction in the role of femininity is supported by the gender literature and in the feminist movement: Some branches of feminism emphasize femininity as a domain of positive distinction from men (e.g., feminism of difference, Gilligan, 1977), while others downplay femininity (Butler, 2002). Thus, in this approach femininity and identification with feminists are not necessarily mutually exclusive.

A further consequence of considering identification with women and feminists as distinct, is that they can have conflicting or opposing effects on attitudes toward certain gender issues, such as when an issue relates to attitudes toward group characteristics *and* group relations. For instance, radical collective action aims to improve the social position of women, and should therefore be positively related to identification with feminists. However, radical collective action may also be negatively related to identification with women, to the extent that radical action is considered gender-atypical behavior for women (Eagly and Steffen, 1986; Hercus, 1999). Additionally, identification with women and identification with feminists may interact in predicting support for radical collective action, so that the positive relationship between identification with feminists and support for radical collective action is stronger amongst women who are less highly committed to typically feminine characteristics (lower identification with women). Likewise, stereotypes are often used to legitimize the intergroup inequality (Jost and Kay, 2005; Rudman and Glick, 2008), and as such endorsement of gender stereotypes is likely to be negatively related to identification with feminists. At the same time, however, gender stereotypes reflect information on what is considered “gender-typical” behavior and can provide differentiation from outgroups (Brewer, 1991; Mlicki and Ellemers, 1996). Thus, when identification with women is low, low attachment to femininity and reduced tendencies to self-stereotype may *strengthen* the effect of identification with feminists on their disapproval of stereotypes.

In sum, in the current paper we propose a multiple identities approach to gender. Importantly, this approach allows both identification with women, and identification with feminists to reflect content, while keeping a simple 2-factor structure. In Study 1, we examine the hypothesis that identification with women and identification with feminists represent separable dimensions of gender identity. We expect that identification with women predicts attitudes toward group characteristics (e.g., femininity) and identification with feminists predicts attitudes toward the social position of the group (e.g., gender inequality). In Studies 2–4, we examine the utility of this multiple identities approach in predicting differences in gender attitudes. Specifically, we expect that identification with women and identification with feminists interact in predicting support for collective action and perceptions of gender stereotypes. All studies reported here were approved by the relevant ethical committees, and conducted in accordance with the Helsinki declaration.

## Study 1

In the first study we examine the central predictions of the multiple identities approach. This study uses a correlational design, to examine the hypothesis that identification with women and identification with feminists will be relatively independent (i.e., not, or only weakly correlated). Secondly, we expect that identification with feminists will predict views on social relations, such as gender equality, and identification with women will predict views on group characteristics, such as perceived femininity.

### Method

#### Participants

Ninety-one female students from the University of Groningen participated in exchange for course credit. The mean age was 20.8 years, ranging from 18 to 48. The majority of participants were German (53%) or Dutch (33%). The remaining 14% indicated another nationality, with 4% indicating non-Western nationalities. Given a multiple regression model with the two identification variables entered as predictors, this sample can detect small-to-medium effect sizes ($R_{\text{change}}^{2}$ ≈ 0.09) with a power of 1 − β = 0.80 when α = 0.05 (G^*^Power, see Faul et al., 2007).

#### Independent variables

##### Identification with women

Identification with women as a group was measured by 4 items (α = 0.77) adapted from Doosje et al. (1995; also see de Lemus et al., 2015). I identify with this group; I have strong ties with this group; This group is an important part of my self-image; Being a member of this group is an important part of how I see myself. These items are easily cast in terms of feminism, allowing us to measure identification with women and identification with feminists with the same items.

##### Identification with feminists

Identification with feminists was measured using the same scale as for identification with women, substituting the word “women” for “feminists” (4 items, α = 0.94).

#### Dependent variables

Each of the measures included in this study used 7-point Likert scales, ranging from “not at all” to “very much”, with the exception of the self-identification measure, which was categorical.

##### Attitudes to group characteristics

*Leach identification scale*. We included the Leach et al. identification scale (Leach et al., 2008, α = 0.87). This scale is composed of five subscales: centrality of group membership, satisfaction with group membership (4 items, e.g., “I am pleased that I am a woman”), solidarity with the group (3 items, e.g., “I feel solidarity with women”), perceived homogeneity of the group (2 items, e.g., “women have a lot in common with each other”), and self-stereotyping (2 items, e.g., “I am similar to the average woman”). Some items of the centrality subscale were also present in the measure of identification with women. Those items were not repeated, and therefore the centrality subscale is not analyzed separately.

*Perceived femininity*. Two items measured perceived femininity of the self: “I am a feminine woman” and “I enjoy doing things that are considered typically feminine” (Leaper and Van, 2008, α = 0.66).

##### Attitudes to group position

*Perceived disadvantage*. Three items (α = 0.65, adapted from Cameron and Lalonde, 2001) were used to create a “perceived disadvantage” scale. These items were “I believe that women are disadvantaged compared to men in today's society,” “If we do nothing, women will continue to be disadvantaged compared to men” and “I have experienced sexism in my daily life.”

*Ambivalent sexism scale*. The ambivalent sexism scale (Glick and Fiske, 1996) consists of the subscales hostile sexism (11 items, α = 0.92), and benevolent sexism (11 items, α = 0.89). The scale includes items such as “Women should be cherished and protected by men” (benevolent), and “Most women interpret innocent remarks as sexist” (hostile).

*Modern sexism scale*. The extent to which people perceive sexism in society was measured by the modern sexism scale (Swim et al., 1995) consisting of 8 items (α = 0.82). The scale includes items such as “Society has reached the point where women and men have equal opportunities for achievement.”

*Attitudes to the feminist movement*. The Attitudes to the Feminist movement Scale (Fassinger, 1994) assesses attitudes toward feminism with items such as “Feminist principles should be adopted everywhere.” The scale consists of 10 items (α = 0.74).

##### Self-identification

The final question asked participants to self-identify as a non-traditional woman, a traditional woman, a feminist or “I don't know” (Gurin and Markus, 1989; Cameron and Lalonde, 2001). This measure was included to distinguish issues related to labeling as a feminist, from issues related to the content of attitudes (Zucker and Bay-Cheng, 2010).

#### Procedure

This study was conducted using Qualtrics. At the start of the questionnaire, participants provided written informed consent and reported demographic information (including gender). Scales were presented in the order described above, items within scales were randomized. It took participants an average of 20 min to complete the study. At the end of the study, participants read a debriefing, and were thanked for their participation.

#### Analytical strategy

Using multiple regression analyses in which identification with women and identification with feminists are simultaneously entered as mean-centered predictors, we examine the hypothesis that identification with women predicts attitudes toward group characteristics, and identification with feminists predicts attitudes toward the social position of the group.

## Results

### Identification with women and feminists

On average women identified strongly with women (*M* = 5.71, *SD* = 0.74; 7-point scale), while identification with feminism was substantially lower (*M* = 3.33, *SD* = 1.38; 7-point scale). The correlation between identification with women and identification with feminists was small (*r* = 0.18, *p* = 0.101), indicating that these are relatively distinct constructs. Given this finding, we examine the content associated with these identities in more detail. Specifically, we hypothesized that identification with women predicts attitudes toward group characteristics (e.g., femininity) and identification with feminists predicts attitudes toward the group's social position (e.g., sexism). The correlations between the different variables are shown in Table 1.

**Table 1:** Correlation table of Study 1.

		**ID with women**	**ID with feminists**	**Femininity**	**Self- stereotyping**	**Solidarity**	**Satisfaction**	**Homogeneity**	**Attitudes to the movement**	**Modern sexism**	**Disadvantage**	**hostile Sexism**
**ID with feminists**	Correlation	0.18	1.000									
	Significance	0.101										
**Femininity**	Correlation	0.62	0.01	1.000								
	Significance	0.000	0.961									
**Self-stereotyping**	Correlation	0.48	0.15	0.52	1.000							
	Significance	0.000	0.167	0.000								
**Solidarity**	Correlation	0.60	0.39	0.40	0.52	1.000						
	Significance	0.000	0.000	0.000	0.000							
**Satisfaction**	Correlation	0.55	0.12	0.50	0.46	0.40	1.000					
	Significance	0.000	0.256	0.000	0.000	0.000						
**Homogeneity**	Correlation	0.11	0.01	0.35	0.48	0.23	0.23	1.000				
	Significance	0.303	0.912	0.001	0.000	0.034	0.032					
**Attitudes to the feminist movement**	Correlation	0.21	0.50	0.10	0.07	0.31	0.14	0.01	1.000			
	Significance	0.055	0.000	0.353	0.541	0.004	0.197	0.914				
**Modern sexism**	Correlation	0.04	0.31	−0.21	−0.39	−0.06	−0.12	−0.31	0.47	1.000		
	Significance	0.719	0.003	0.059	0.000	0.578	0.262	0.014	0.000			
**Disadvantage**	Correlation	−0.05	0.42	−0.13	−0.29	0.04	−0.21	−0.11	0.32	0.56	1.000	
	Significance	0.654	0.000	0.218	0.006	0.740	0.056	0.299	0.003	0.000		
**Hostile sexism**	Correlation	−0.17	−0.26	0.02	−0.01	−0.07	−0.02	0.18	−0.47	−0.44	−0.28	1.000
	Significance	0.123	0.017	0.848	0.945	0.533	0.858	0.097	0.000	0.000	0.010	
**Benevolent sexism**	Correlation	0.06	0.07	0.19	0.24	0.20	0.16	0.25	−0.14	−0.38	−0.14	0.57
	Significance	0.580	0.523	0.086	0.067	0.067	0.151	0.020	0.155	0.000	0.211	0.000

#### Hypothesis test

##### Attitudes toward group characteristics

In line with our hypothesis, attitudes related to group characteristics were predicted by identification with women, but not identification with feminists (see Table 2). Specifically, higher identification with women was associated with higher self-rated femininity [*B* = 0.24, *SE* = 0.03, *t*_(88)_ = 7.42, *p* < 0.001, $R_{\text{change}}^{2}$ = 0.40]. Those who were more strongly identified with women were also more likely to self-stereotype [*B* = 0.21, *SE* = 0.04, *t*_(88)_ = 4.77, *p* < 0.001, $R_{\text{change}}^{2}$ = 0.21] and more satisfied with being a group member [*B* = 0.62, *SE* = 0.11, *t*_(88)_ = 5.79, *p* < 0.001, $R_{\text{change}}^{2}$ = 0.28]^1^.

**Table 2:** Attitudes predicted by identification with women, and identification with feminists in Study 1.

**Dependent variable**	**Predictor**	***B***	***SE***	***t*-value**	***p*-value**	**$R_{\text{change}}^{2}$**
Femininity	Identification with women	0.24	0.032	*t* = 7.42	*p* < 0.001	0.40
	Identification with feminists	−0.02	0.017	*t* = −1.26	*p* = 0.212	0.01
Self-stereotyping	Identification with women	0.21	0.044	*t* = 4.77	*p* < 0.001	0.21
	Identification with feminists	0.02	0.24	*t* < 1	*p* = 0.492	0.004
Satisfaction	Identification with women	0.62	0.11	*t* = 5.79	*p* < 0.001	0.28
	Identification with feminists	0.02	0.06	*t* < 1	*p* = 0.769	0.0007
modern sexism	Identification with women	−0.04	0.24	*t* < 1	*p* = 0.873	0.0003
	Identification with feminists	0.39	0.13	*t* = 2.991	*p* = 0.004	0.10
Perceived disadvantage	Identification with women	−0.08	0.06	*t* = −1.27	*p* = 0.209	0.016
	Identification with feminists	0.15	0.03	*t* = 4.38	*p* < 0.001	0.19
Hostile sexism	Identification with women	−0.05	0.04	*t* = −1.21	*p* = 0.231	0.016
	Identification with feminists	−0.05	0.02	*t* = −2.2	*p* = 0.031	0.05
Benevolent sexism	Identification with women	0.02	0.04	*t* < 1	*p* = 0.651	0.003
	Identification with feminists	0.01	0.02	*t* < 1	*p* = 0.580	0.003
Attitudes to feminist movement	Identification with women	0.02	0.2	*t* = 1.28	*p* = 0.206	0.01
	Identification with feminists	0.05	0.01	*t* = 5.05	*p* < 0.001	0.23
Solidarity with women	Identification with women	0.18	0.03	*t* = 6.64	*p* < 0.001	0.30
	Identification with feminists	0.05	0.01	*t* = 3.46	*p* = 0.001	0.08

##### Attitudes toward the group's social position

Further, as hypothesized, attitudes related to the group's social position were predicted by identification with feminists, but not identification with women (see Table 2). Specifically, higher identification with feminists was related to increased perceptions of modern sexism [*B* = 0.39, *SE* = 0.13, *t*_(88)_ = 2.99, *p* = 0.004, $R_{\text{change}}^{2}$ = 0.10]. Likewise, high identification with feminists was associated with higher perceptions of disadvantage for women [*B* = 0.15, *SE* = 0.03, *t*_(88)_ = 4.38, *p* = 0.001, $R_{\text{change}}^{2}$ = 0.19] and endorsed less hostile sexism [*B* = −0.05, *SE* = 0.02, *t*_(88)_ = −2.20, *p* = 0.031, $R_{\text{change}}^{2}$ = 0.05]. Note, however, that the effect of identification with feminists on hostile sexism is quite small given the size of the sample used in this study. Finally, as would be expected, identification with feminists predicted more positive attitudes to the feminist movement, *B* = 0.05, *SE* = 0.01, *t*(88) = 5.05, *p* < 0.001, $R_{\text{change}}^{2}$ = 0.23^2^.

Solidarity with the group was predicted by both identification with feminists [*B* = 0.05, *SE* = 0.01, *t*_(88)_ = 3.46, *p* = 0.001, $R_{\text{change}}^{2}$ = 0.08] and identification with women [*B* = 0.18, *SE* = 0.02, *t*_(88)_ = 6.64, *p* < 0.001, $R_{\text{change}}^{2}$ = 0.30], such that solidarity with women as a group was highest amongst those who identified strongly with *both* women and feminists. Endorsement of benevolent sexism, and perceived homogeneity of the group were not affected by either identification with women or feminists (*t*s < 1).

#### Additional measures

The measure of self-report identification showed that 49% of the participants identified themselves as non-traditional women, 18% indicated that they thought of themselves as traditional women, only a very small percentage (5%) identified as feminists, and 28% indicated that they did not know. Thus, more than a quarter of women could not or would not classify themselves. Although the percentage of women explicitly identifying as feminists was very small (5%), identification with feminists distinguished those who self-labeled as feminists from those who did not [χ^2^(3) = 14.36, *p* = 0.002]. Importantly, the different self-identification categories could not reliably predict attitudes toward gender issues (femininity, satisfaction, modern sexism, and disadvantage) (Wald's *Z* < 1.37, *p*s > 0.241). That is, correspondence between categorical self-identification and attitudes toward gender issues is limited, confirming the discrepancy noted by previous work (Zucker and Bay-Cheng, 2010).

### Discussion

In this study, identification with feminists and identification with women showed only a small correlation (consistent with Roy et al., 2007). Moreover, there was evidence that identification with women correlates with attitudes toward group characteristics, and identification with feminists correlates with attitudes to the group's social position. These findings support predictions of the multiple identities approach which permits content for both identities. The difference between the identities is the *type* of content they incorporate.

Results of this study confirmed the relative independence of the two identities, suggesting that identification as a feminist can exist alongside a sense of personal femininity, a pattern reflected in high identification with women *and* feminists. These women also showed the highest solidarity with the broader group of women. At first sight, the combination between satisfaction with group membership associated with identification with women, and perceptions of disadvantage associated with identification with feminists, may seem contradictory. However, these concerns may be reconciled by a desire to accord more status and value to typically feminine attributes, tasks and interests: maintaining a focus on femininity, while at the same time resolving disadvantage. In fact, it could be argued that if feminism implies defending the notion that femininity is not inferior to masculinity, then feminism does not undermine femininity, but rather affirms it.

It is worth noting that in this study, only a very small number of women (4%) self-labeled as feminists. This finding is in line with findings of previous research showing that women are reluctant to self-identify as feminists, even though they may hold feminist attitudes (Aronson, 2003; Zucker and Bay-Cheng, 2010). Such under-use of one category means that only the remaining three categories are used to self-categorize, which limits the variance of such categorical measures and suggests that it is preferable to measure identification with women and feminists as continuous variables.

Further, it is noteworthy that in this study, the mean of identification with women is above the mid-point of the scale, which means that “low identification with women” in this study is relative, rather than absolute. Indeed, there may be many different issues that affect the absolute mean levels of identification in a certain sample. For instance, making salient inter-group competition can increase levels of in-group identification reported (David and Turner, 1999). More specific to the gender context, however, the finding that the mean of identification with women is above the mid-point of the scale might be explained, in part, by the fact that belonging to the category of women is not purely chosen, but “ascribed” by others (based, largely, on biological indicators). In other words, in the case of identification with women, even a woman who is dissatisfied with her group membership and does not consider herself typical of the group, likely still considers herself a woman. Identification with feminists, on the other hand, is more similar to a chosen identity or an opinion-based group (Bliuc et al., 2007; McGarty et al., 2009). Such considerations might inspire a “baseline” level of identification with women, as reflected in somewhat higher overall means.

Taken together, results of Study 1 suggested a relatively clear-cut division of attitudes as either relating to group characteristics *or* the group's social position. However, many gender issues are more complex than this, and have implications for group characteristics as well as the group's social position. In such a case, we may expect both identification with women and identification with feminists to play a role in determining attitudes to such an issue, through additive or interactive effects. Studies 2–4 further explore the utility of the multiple identities approach in predicting attitudes to gender issues that may relate to concern for group characteristics as well as concern for the group's social position.

## Study 2

In Study 2, we examine the utility of the multiple identity approach in predicting attitudes to gender issues that have a bearing both on concern for group characteristics and the group's social position, focusing specifically on collective action. Collective action is aimed at confronting disadvantage and producing social change (Van Zomeren and Iyer, 2009), and in the current study we distinguish between radical and moderate forms of collective action (Tausch et al., 2011).

In the context of gender, it has been shown that identification with feminists has a positive relationship with collective action (Liss et al., 2004; Nelson et al., 2008; Yoder et al., 2011). Those who identify strongly with feminism perceive that women are disadvantaged in society, and as such they wish to change the status quo. When considering identification with women, there is reason to expect that it will not have a strong relationship with collective action, as collective action does not relate directly to group characteristics. Thus, we expect that high identification with women does not necessarily lead to increased support for collective action (Henderson-King and Stewart, 1994). However, in the case of radical collective action, we may expect that identification with women will have a *negative* effect on support for this type of action. Radical collective action is often defined as collective actions that involve some degree of aggression, anger, or even violence (Tausch et al., 2011), traits that are oppositional to social definitions of femininity (Eagly and Steffen, 1986; Hercus, 1999; Fiske et al., 2002). Based on this line of reasoning, we might also expect an interaction between identification with women and identification with feminists when considering radical collective action. Only women who are strongly identified with feminists are likely to consider radical action to improve the social position of women, meaning that support for radical action is low when identification with feminists is low, irrespective of identification with women. However, the motivating influence of identifying as a feminist for radical action will only lead to actual support amongst those who are relatively unconcerned about radical action being atypical and uncharacteristic for the group (i.e., when identification with women is low). That is, higher identification with women might dampen the effect of strong identification with feminists on support for radical action. If this is the case, we would expect support for radical action only amongst those women who identify with feminists but not women. Study 2 examines this possibility.

In sum, using a correlational design, this study examines the hypothesis that support for collective action is affected by both identification with women, and identification with feminists. We expect that support for (radical and moderate) collective action is stronger amongst those who are more highly identified with feminists, but less so with women.

### Method

#### Participants

One hundred and twenty one female participants were recruited amongst students of the University of Granada, Spain. Age ranged from 18 years old to 50 years old, with an average of 19.75. Participants took part in exchange for course credit. Given a multiple regression model with the two identification variables and their interaction entered as predictors, this sample can detect small-to-medium effect sizes ($R_{\text{change}}^{2}$ ≈ 0.065) with a power of 1 −β = 0.80 when α = 0.05 (G^*^Power, see Faul et al., 2007).

#### Design

Data for this study were collected as part of a larger experiment (de Lemus et al., in preparation) with a 2 × 2 between-participants design. Identification with women and identification with feminists were measured alongside the manipulated factors, and the effect of the identification variables on support for collective action is the focus of the current study. As such, this study uses a correlational design.

##### Manipulation

Data for this study were collected as part of a larger experiment which included a 2 × 2 between-participants manipulation. The first manipulated factor exposed participants to either stereotypical or counter-stereotypical gender roles through pictures showing men and women in kitchen or office settings. Stimuli for this exposure phase were pictures of men and women, appearing in three different contexts (kitchen, office, and a neutral outdoor setting). The same 6 persons (3 women and 3 men), with an emotionally neutral face, appeared in the different contexts. Participants in the stereotype condition were presented with 90% of the women appearing in a kitchen, and 90% of the men appearing in an office; whereas those in the counter-stereotype condition were presented with 90% of the men appearing in a kitchen, and 90% of the women appearing in an office (counter-stereotypical exposure group). Interspersed with the (counter-) stereotypical pictures were neutral trials (*N* = 16) in which men and women appeared outdoors. Participants were presented with 160 trials in total during the exposure phase.

After the exposure phase participants completed an evaluative decision task. Participants were required to classify target words as either positive or negative. Each target word was preceded by a picture prime. The primes used were the second manipulated factor: Half of the participants saw stereotypical gender roles as primes, whereas the other half of the participants completed the task with male and female faces as primes. That is, in both cases the primes conveyed gender information, but for half the participants the primes also invoked gender role information. The evaluative decision task consisted of 4 blocks of 64 trials.

These two manipulated factors created 4 experimental between-participants conditions: stereotype exposure and faces primes, stereotype exposure and role primes, counter-stereotype exposure and faces primes, counter-stereotype exposure and role primes. Crucially for the current study, however, the manipulated factors did not affect support for collective action, either on their own, or in interaction with the identification variables, as described in the “analytical strategy” section below.

#### Independent variables

##### Identification with women and identification with feminists

Identification with women and identification with feminists were measured in the same way as in Study 1 (4 items each; α = 0.78 and α = 0.95, respectively).

#### Dependent variables

A complete list of the dependent variables included can be found in the Supplementary Materials. Below we describe only the measures of interest for this study.

##### Support for collective action

Support for moderate collective action was measured by 6 items (α = 0.68), focusing on actions like signing a petition, joining a peaceful public demonstration, or lobbying for women's rights. Support for radical collective action was measured with 5 items (α = 0.76), focusing on actions like attacks on sexist institutions, blackmailing, or hacking into e-mail accounts (Tausch et al., 2011). Support for each action was rated on an 11-point scale from not at all to very much. All items referred to the action being taken in order to “reduce gender inequality.” Thus, it was clear that the objective of both types of action was the same, only the form differed.

##### Perceived efficacy

Perceived efficacy of women as a group was measured with three items (α = 0.82) adapted from van Zomeren et al. (2008). The scale includes such items as “Together, women can achieve their aims.” This was used as a control variable in the analyses.

#### Procedure

Participants provided written informed consent, were assigned to one of four conditions, and completed the manipulation. Participants then completed a paper-and-pencil questionnaire, with the measures of central interest, identification with feminists, identification with women and support for collective action at the end. After completing all measures, participants read a funneled debriefing and were thanked for their participation.

#### Analytical strategy

Because the measures of interest in this study were taken after a manipulation we examined the effect of the manipulated factors on identification with women, identification with feminists and collective action intentions, but no effects were found (*F*s < 2.5, *p* > 0.116). However, identification with women [*t*_(120)_ = 4.77, *p* < 0.001], identification with feminists [*t*_(120)_ = 4.66, *p* < 0.001] and support for moderate collective action [*t*_(120)_ = 5.02, *p* < 0.001] were all related to perceived group efficacy. Therefore, group efficacy is controlled for in the analyses presented below.

Using multiple regression analysis, we examine the hypothesis that both identification with feminists and identification with women affect support for collective action. Specifically with regards to radical collective action, we expect an interaction between identification with women and identification with feminists. Therefore, identification with feminists, identification with women, and their interaction are entered into the regression model as mean-centered predictors.

### Results

#### Identification with women and feminists

As in Study 1, participants identified strongly with women as a group (*M* = 5.82; *SD* = 0.88), and less with feminists (*M* = 3.63; *SD* = 1.58). Again, identification with feminists and identification with women were not significantly correlated (*r* = 0.12, *p* = 0.193). The correlations between the different variables are shown in Table 3.

**Table 3:** Correlation table for Study 2.

**Control variable**			**ID with women**	**ID with feminists**	**Moderate action**
Efficacy	ID with feminists	Correlation	0.12	1.000	
		Significance	0.193		
	Moderate action	Correlation	0.13	0.26	1.000
		Significance	0.143	0.004	
	Radical action	Correlation	−0.10	0.34	0.22
		Significance	0.261	0.000	0.013

#### Hypothesis test

Moderate and radical collective action were weakly but significantly related (*r* = 0.22, *p* = 0.013). Support for moderate action was higher (*M* = 8.28) than support for radical action (*M* = 2.46). Support for moderate collective action was predicted by identification with feminists [*B* = 0.21, *SE* = 0.08, *t*_(120)_ = 2.73, *p* = 0.007, $R_{\text{change}}^{2}$ = 0.05]: those who identified more strongly with feminists were more likely to support moderate collective action. There was no effect of identification with women on support for moderate collective action (*t* < 1.31). Support for radical collective action was positively predicted by identification with feminists [*B* = 0.35, *SE* = 0.09, *t*_(120)_ = 4.01, *p* < 0.001, $R_{\text{change}}^{2}$ = 0.12], while identification with women *negatively* predicted support for radical action [*B* = −0.34, *SE* = 0.15, *t*_(120)_ = −2.20, *p* = 0.030, $R_{\text{change}}^{2}$ = 0.035], though this effect was small in size. The interaction between identification with women and identification with feminists did not reach significance (*t* < 1.24, *p* > 0.218)^3^. These effects illustrate that support for radical collective action is higher when identification with feminists is high, and identification with women is low. This pattern was the result of additive effects rather than an interaction, and as such provides partial support for our hypothesis.

### Discussion

This study replicates findings from Study 1 that identification with women and identification with feminists constitute separable dimensions of gender identity. Additionally, results from this study show that those who identify more strongly with feminists are more likely to support both moderate and radical collective action strategies aimed at increasing equality between the groups. This is in line with results from Study 1, which suggests that identification with feminists is related to attitudes toward the group's social position (inequality, sexism, relative status). Identification with women on the other hand did not predict support for moderate collective action, and there was some evidence that it *negatively* predicted support for radical collective action. This indicates that high identification with women does not automatically translate to increased support for collective action, and when collective action seems to contradict social definitions of femininity (radical), higher identification with women is associated with somewhat *reduced* support for such actions. In addition to these additive effects we also considered the possibility of an interaction between identification with women and identification with feminists, but there was no evidence for this.

In sum, Study 2 shows that support for moderate collective action increases with identification with feminists, but is not related to identification with women. Support for radical collective action is highest amongst those women who identify strongly with feminists but not women, due to additive effects of identification with women and identification with feminists.

## Study 3

Study 3 examines another domain expected to relate to both identification with women and identification with feminists: gender stereotypes. Study 1 showed that identification with feminists is related to concern for the societal position of women. As stereotypes are often used to legitimize the gender hierarchy (Jost and Kay, 2005; Rudman and Glick, 2008) they can be seen as unfair and disadvantageous for women. Therefore, it is likely that those who are strongly identified with feminists find gender stereotypes more problematic than those who are less strongly identified with feminists. At the same time, gender stereotypes provide information about which behaviors are considered typical and appropriate for the group (Prentice and Carranza, 2002), and provide a basis for differentiation from out-groups (Spears et al., 1997), in this case, men. Given that Study 1 showed that identification with women is related to attitudes toward group characteristics, it is likely that those who are strongly identified with women find gender stereotypes *less* problematic than those for whom identification with women is lower. Thus, we might expect additive effects of identification with women and identification with feminists on perceptions of gender stereotypes. However, we might also expect identification with women and identification with feminists to interact. Specifically, we argue that the effect of identification with feminists on critical attitudes toward gender stereotypes will be stronger amongst those for whom group characteristics are less important to their identity (lower identification with women). In other words, because those who are less strongly identified with women attach less importance to typical group characteristics and attributes, identification with feminists more easily leads to criticism of gender stereotypes, because there is no conflict between the two motivations. This line of reasoning suggests that identification with feminists leads to critical attitudes toward stereotypes, particularly for lower levels of identification with women.

This study uses an experimental design to examine the hypothesis that attitudes toward gender stereotypes are predicted by identification with women, identification with feminists, and their interaction. Identification with women and feminists are measured continuously, as in Studies 1 and 2. Attitudes toward gender stereotypes are assessed with a direct self-report measure, as well as an indirect measure. The indirect measure of attitudes toward gender stereotypes exposes participants to a (within-participants) manipulation in which two women express different views of gender stereotypes: one speaker is critical of gender stereotypes, while the other speaker endorses gender stereotypes. The dimension of interest is differences in participants' agreement with one speaker over the other.

### Method

#### Participants

A community sample of 201 female participants was recruited through ProlificAcademic. Of these, 59% were from the United Kingdom, 37% were from the United States, and 4% had other nationalities. Age ranged from 16 years old to 68 years old, with a mean age of 30.6 (*SD* = 10.758 years). Eight participants were excluded because their completion times exceeded the mean completion time by more than 3 SD, indicating that they had not completed the study in one sitting. Six participants were excluded because they failed the attention check. Three further participants indicated that they had trouble understanding the questions, and were also excluded. The final sample included 184 participants. Given a multiple regression model with the two identification variables and their interaction entered as predictors, this sample can detect small-to-medium effect sizes ($R_{\text{change}}^{2}$ ≈ 0.043) with a power of 1 − β = 0.80 when α = 0.05 (G^*^Power, see Faul et al., 2007).

#### Independent variables

##### Identification with women and identification with feminists

Identification with women and identification with feminists were measured in the same way as the previous studies (α = 0.87 and α = 0.97, respectively).

##### Manipulation

We created a within-participants manipulation that presented participants with a conversation between two women. The manipulated factor is the attitudes expressed by each of these women: one speaker criticizes gender stereotypes, the other endorses them. Each speaker made 2 arguments. The anti-stereotype speaker argues that stereotypes are problematic because they legitimize and exacerbate disadvantage faced by women. The pro-stereotype speaker argues that stereotypes in themselves are not always negative. Thus, we created a within-participants manipulation with 2 levels (anti-stereotype vs pro-stereotype). As a dependent variable we then measured the extent to which our participants agreed with each of the speakers (see details below). In sum, this measure was designed as an indirect measure of participants' views of gender stereotypes.

#### Dependent variables

##### Ratings of speakers

After reading the manipulation, participants rated the speakers on how much they agreed with them, how considerate, friendly and intelligent they found them, and how much they liked them. Ratings on these dimensions were highly correlated (*rs* > 0.7) and taken together as an indicator of participants' positive attitudes toward the speaker. We expected that ratings of the speakers would be affected by the interaction between identification with women and identification with feminists, such that higher identification with feminists leads to a preference for the anti-stereotype speaker over the pro-stereotype speaker, and that this relationship becomes stronger for lower levels of identification with women. This measure was analyzed with multiple regression analysis. As our hypotheses focus on preferences for one speaker over the other, we created a difference score reflecting differences in ratings of the speakers by subtracting ratings of the anti-stereotype speaker from ratings of the pro-stereotype speaker. That is, a positive difference score represents a preference for the pro-stereotype speaker, and a negative difference score represents a preference for the anti-stereotype speaker. The analysis focused on predicting these differences between the ratings of the two speakers from the identification variables, and their interaction^4^.

##### Perceptions of stereotypes

As a second, more direct, measure of perceptions of stereotypes, participants saw a list of pre-tested statements reflecting descriptive (*N* = 10, α = 0.93), and prescriptive stereotypes of women (*N* = 4, α = 0.91) (Rudman, 1998; Eagly and Karau, 2002). Examples included “women are less aggressive than men” (descriptive), and “women should be more caring than men” (prescriptive). For each of these items, participants rated how problematic they found the statement. Preliminary analyses revealed that participants found prescriptive stereotypes significantly more problematic than descriptive stereotypes [*M*_difference_ = 1.31, *t*_(183)_ = 13.16, *p* < 0.001] and therefore descriptive and prescriptive items were analyzed separately. This measure was analyzed with multiple regression analyses in which identification with women, identification with feminists, and their interaction were entered as predictors. We expected that the identification variables will interact, such that women find stereotypes more problematic when they are more strongly identified with feminists, and that this relationship is stronger for lower levels of identification with women.

In addition to these central measures, we included measures of perceived femininity of the self (α = 0.87), perceived disadvantage for women (α = 0.93), Modern Sexism (α = 0.77), hostile sexism (α = 0.94), and benevolent sexism (α = 0.92). These measures were included to replicate findings of Study 1, and they were measured as described above. Finally, some exploratory measures were included, which are described in the Supplementary Materials.

#### Procedure

Data was collected through Qualtrics. Participants accessed the study through the ProlificAcademic website. At the start of the study, participants provided written informed consent, completed demographic information (including gender), as well as the measures of identification with feminists and identification with women, and the replication measures. They then read the manipulation text and rated the speakers and arguments, followed by the measure of attitudes toward stereotypes. At the end of the study, participants read a debriefing and were thanked for their participation.

#### Analytical strategy

We assess the correspondence between findings of this study and those of Study 1 using multiple regression analysis. Predictors are identification with women, and identification with feminists. When evaluating our hypotheses regarding the effects of the manipulation, and perceptions of gender stereotypes we include the interaction between identification with women and identification with feminists in the multiple regression model as a third predictor.

### Results

#### Identification with women and feminists

Identification with women was above the mid-point of the scale (*M* = 4.93, *SD* = 0.91; 7-point scale), while identification with feminists was below the mid-point of the scale (*M* = 3.37, *SD* = 1.53; 7-point scale). The correlation between identification with women and identification with feminists was somewhat higher than in previous studies, *r* = 0.25, and this correlation was significant (*p* = 0.001). Table 4 shows the correlations between the different variables.

**Table 4:** Correlation table for Study 3.

		**ID with Women**	**ID with feminists**	**Femininity**	**Perceived Disadvantage**	**Modern Sexism**	**Hostile Sexism**	**Benevolent Sexism**	**Descriptive-Problematic**	**Prescriptive-Problematic**	**Anti Speaker**
**ID with feminists**	Correlation	0.25	1								
	Significance	0.000									
**Femininity**	Correlation	0.51	0.11	1							
	Significance	0.000	0.142								
**Perceived disadvantage**	Correlation	0.20	0.41	0.05	1						
	Significance	0.006	0.000	0.508							
**Modern sexism**	Correlation	0.13	0.66	−0.09	0.55	1					
	Significance	0.085	0.000	0.251	0.000						
**Hostile sexism**	Correlation	−0.004	−0.49	0.14	−0.33	−0.59	1				
	Significance	0.952	0.000	0.056	0.000	0.000					
**Benevolent sexism**	Correlation	0.36	−0.12	0.31	−0.10	−0.21	0.46	1			
	Significance	0.000	0.098	0.000	0.182	0.004	0.000				
**Descriptive-problematic**	Correlation	−0.13	0.33	−0.18	0.20	0.27	−0.36	−0.39	1		
	Significance	0.071	0.000	0.014	0.006	0.000	0.000	0.000			
**Prescriptive -problematic**	Correlation	−0.12	0.26	−0.13	0.34	0.32	−0.34	−0.48	0.48	1	
	Significance	0.116	0.000	0.089	0.000	0.000	0.000	0.000	0.000		
**Anti speaker**	Correlation	0.14	0.26	0.03	0.12	0.23	−0.10	0.05	0.1	−0.004	1
	Significance	0.055	0.000	0.665	0.099	0.002	0.199	0.504	0.164	0.952	
**Pro speaker**	Correlation	0.41	0.19	0.24	0.15	0.21	−0.08	0.15	0.02	−0.01	0.23
	Significance	0.000	0.010	0.001	0.040	0.003	0.292	0.039	0.815	0.876	0.001

#### Correspondence with study 1

Overall, findings of this study correspond largely to the results of Study 1. Like in Study 1, identification with women predicted attitudes toward group characteristics, and identification with feminists predicted attitudes toward the social position of the group. The statistical information for these findings is presented in Table 5. Specifically, as in Study 1, those who were more strongly identified with women saw themselves as more feminine than those who were less strongly identified with women. Moreover, as in Study 1, stronger identification with feminists was associated with higher perceptions of modern sexism in society, and disadvantage for women as a group, as well as reduced endorsement of hostile sexism. Benevolent sexism was predicted by additive effects of identification with women and identification with feminists. Stronger identification with feminists was associated with lower endorsement of benevolent sexism, while stronger identification with women was associated with *higher* endorsement of benevolent sexism.

**Table 5:** Attitudes predicted by identification with women, and identification with feminists in Study 3.

**Dependent**	**Predictor**	***B***	***SE***	***t*-value**	***p*-value**	**$R_{\text{change}}^{2}$**
Femininity	Identification with women	0.66	0.08	*t* = 7.95	*p* < 0.001	0.25
	Identification with feminists	−0.02	0.05	*t* < 1	*p* = 0.713	0.0005
Modern sexism	Identification with women	−0.03	0.05	*t* < 1	*p* = 0.533	0.001
	Identification with feminists	0.34	0.03	*t* = 11.75	*p* < 0.001	0.42
Perceived disadvantage	Identification with women	0.07	0.05	*t* = 1.45	*p* = 0.148	0.009
	Identification with feminists	0.18	0.03	*t* = 5.65	*p* < 0.001	0.14
Hostile sexism	Identification with women	0.15	0.08	*t* = 1.82	*p* = 0.070	0.01
	Identification with feminists	−0.36	0.05	*t* = −7.79	*p* < 0.001	0.25
Benevolent sexism	Identification with women	0.49	0.08	*t* = 5.97	*p* < 0.001	0.16
	Identification with feminists	−0.15	0.05	*t* = −3.21	*p* = 0.002	0.05

#### Hypothesis test

##### Effects of the manipulation

The manipulation exposed participants to an anti-stereotype speaker and a pro-stereotype speaker. Overall, the pro-stereotype speaker was given more positive ratings than the anti-stereotype speaker [*M*_*difference*_ = 0.61, *t*_(183)_ = 7.60, *p* < 0.001]. The preference for the pro-stereotype speaker over the anti-stereotype speaker was particularly strong amongst those who are highly identified with women [*B* = 0.30, *SE* = 0.09, *t*_(183)_ = 3.21, *p* = 0.002, $R_{\text{change}}^{2}$ = 0.054]^5^. That is, those who are highly identified with women rated the pro-stereotype speaker more positively than the anti-stereotype speaker. However, there was no evidence that ratings of the speakers were affected by the interaction between identification with women and identification with feminists (*t* < 1), and as such our hypothesis was not supported.

##### Perceptions of stereotypes

**Figure 1:**
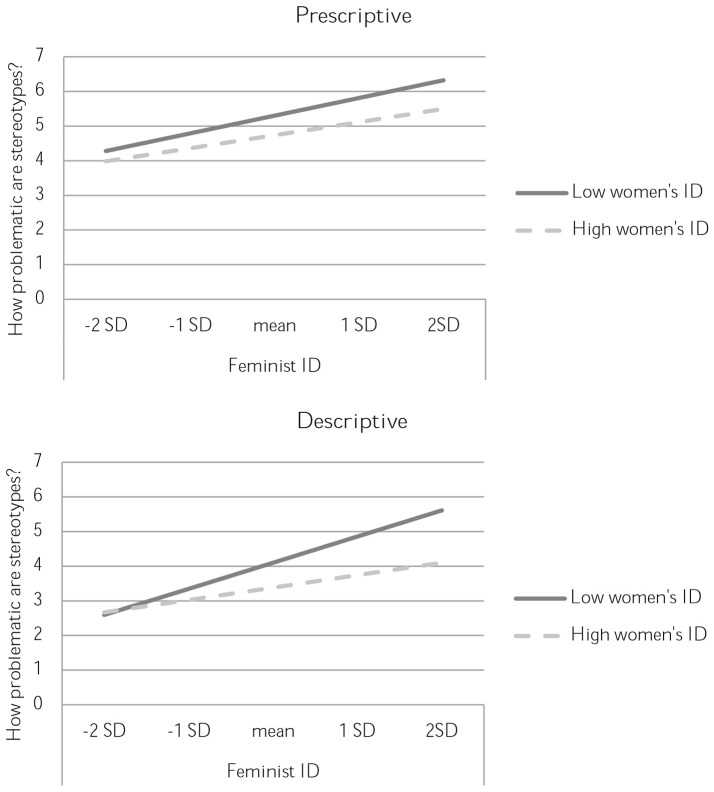
Perceptions of the problematic nature of stereotypes in Study 3, separated by prescriptive **(top)** vs. descriptive **(bottom)** phrasing. High and low women' s identification are plotted at ±1 standard deviation from the mean.

Participants indicated how problematic they found prescriptive and descriptive stereotypes of women. Results are depicted in Figure 1.

For prescriptive stereotypes (see Figure 1 top), there were additive main effects of identification with feminists and identification with women. Prescriptive stereotypes are perceived as more problematic at higher levels of identification with feminists, *B* = 0.28, *SE* = 0.07, *t*_(183)_ = 4.25, *p* < 0.001, $R_{\text{change}}^{2}$ = 0.09. Prescriptive stereotypes are perceived as less problematic at higher levels of women's identification [*B* = −0.28, *SE* = 0.11, *t*_(183)_ = −2.56, *p* = 0.011, $R_{\text{change}}^{2}$ = 0.03].

For descriptive stereotypes, there was an interaction between identification with women and identification with feminists [*B* = −0.13, *SE* = 0.06, *t*_(183)_ = −2.05, *p* = 0.042, $R_{\text{change}}^{2}$ = 0.02]. Decomposition of the interaction showed that women who are more strongly identified with feminists are more critical of gender stereotypes [*B* = 0.35, *SE* = 0.06, *t*_(183)_ = 5.78, *p* < 0.001, $R_{\text{change}}^{2}$ = 0.15]. This effect of identification with feminists is stronger when identification with women is low, [*B* = 0.47, *SE* = 0.09, *t*_(183)_ = 5.09, *p* < 0.001, $R_{\text{change}}^{2}$ = 0.12] than when identification with women is high [*B* = 0.13, *SE* = 0.08, *t*_(183)_ = 1.53, *p* = 0.128, $R_{\text{change}}^{2}$ = 0.01]. This effect is depicted in the Figure 1 bottom. An alternative breakdown of the interaction revealed another significant simple slope which showed that, when identification with feminists is high, identification with women has a dampening effect on critical attitudes to gender stereotypes [*B* = −0.52, *SE* = 0.14, *t*_(183)_ = −3.76, *p* < 0.001, $R_{\text{change}}^{2}$ = 0.06], an effect that is not present when identification with feminists is low (*t* < 1). In Figure 1 (bottom panel), this effect is evident from the fact that the lines representing lower vs. higher identification with women diverge more strongly at higher levels of identification with feminists.

These findings support the hypothesis that the interaction between identification with women and identification with feminists predict critical attitudes toward descriptive gender stereotypes. Those who are highly identified with feminists but not women are particularly likely to consider stereotypes problematic. For prescriptive stereotypes, a similar pattern appeared as a result of additive effects.

### Discussion

Study 3 replicated results from Study 1 in a community sample. Identification with women was related to attitudes toward group characteristics, while identification with feminists was related to attitudes regarding the group's position. These findings support the multiple identities approach in showing that identification with women and identification with feminists are distinguishable components of gender identity.

Aside from replicating earlier studies, this study also showed some novel findings. Specifically, in line with our hypothesis, results showed that women find gender stereotypes more problematic at higher levels of identification with feminists, and *lower* levels of identification with women. This pattern appeared as a result of additive effects for prescriptive stereotypes, and as an interaction for descriptive stereotypes. These findings illustrate that, in line with the multiple identities reasoning, different combinations of the two identities can lead to distinct gender attitudes. Given these findings, it is worth noting that the fact that some women do not object to (some forms of) stereotypes need not mean that they accept the lower status implications associated with gender stereotypes. They may support the notion of “typically female” activities and interests, but still object to the idea that these imply lower status. For instance, they may argue that typically female traits such as warmth should be valued more.

Interestingly, several other recent studies have found evidence for interactive effects of identification with women and feminists on gender attitudes (e.g., in this issue Leicht et al., 2017; van Breen et al., in preparation). One way of understanding such interactions, and conceptualizing how the two identities may be combined, is by thinking of the different combinations as theoretical “identity types” or identity profiles. In such a taxonomy, the first group includes women who are not strongly identified with either women or feminists (and as such are relatively “low identifiers”). Secondly, there are those who identify strongly with women but not feminists (“traditional women”; see Condor, 1986). In addition, there are two feminist profiles: those who are highly identified with feminists *and* women (whom we might call “dual identifiers”, see in this issue Leicht et al., 2017), and those who identify strongly with feminists, but less strongly with women (whom we refer to here as “distinctive feminists”). These different identification “profiles” are not necessarily fixed or absolute categories, but rather should be seen as a way of conceptualizing different approaches to integrating the identities. We return to this conceptualization in more detail in the General Discussion.

Although the interaction was in line with our hypotheses and findings from other studies, the small size of the effect means that more power is needed to assess its reliability. Therefore, Study 4 will aim to replicate the interaction. One additional limitation of the current study is that the measure of perceptions of stereotypes asked only about how problematic participants found the statements. Participants might find certain statements problematic for different reasons. For instance, they may find stereotypes problematic because they are untrue, but they may also consider stereotypes problematic precisely because they *are* true. Additionally, it is worth noting that the manipulation of perceptions of stereotypes did not produce the expected effects. Study 4 examines these issues in more detail.

## Study 4

This study aimed, firstly, to replicate the findings of Study 3, and to refine the measure of perceptions of stereotypes. While Study 3 asked only how problematic participants found the statement presented, the current study also asked how *true* participants found the statements. In all other respects, the design and measures of Study 4 were identical to those of Study 3. Based on the results of Study 3, we expect that women will find gender stereotypes more problematic at higher levels of identification with feminists, and lower levels of identification with women—as a result of interactive effects in the case of descriptive stereotypes, and as a result of additive effects in the case of prescriptive stereotypes.

Like Study 3, this study uses an experimental design to examine the hypothesis that attitudes toward gender stereotypes are predicted by the interaction between identification with women and identification with feminists. The experimental factor consists of a within-participants manipulation that exposes participants to a scenario in which two women discuss different views to gender stereotypes. Identification with women and feminists are measured on a continuous scale.

### Method

#### Participants

Participants were 200 female students at the University of Groningen. Age ranged from 17 years old to 31 years old, with a mean age of 19.7 (*SD* = 2.08). Three participants were excluded because they failed the attention checks. One participant had to be excluded because she completed the study twice. The final sample included 196 participants. Given a multiple regression model with the two identification variables and their interaction entered as predictors, this sample can detect small-to-medium effect sizes in the range of those found in Study 3 ($R_{\text{change}}^{2}$ ≈ 0.04) with a power of 1 − β = 0.80, when α = 0.05 (G^*^Power, see Faul et al., 2007).

#### Independent variables

The independent variables were the same as in Study 3: identification with women, identification with feminists and the within-participants manipulation.

#### Dependent variables

The dependent variables in this study were largely the same as in Study 3. Only those measures that were added or adapted are described below.

##### Perceptions of stereotypes

As in Study 3, we examined women's attitudes toward stereotypes indirectly through participants' evaluation of the conversation between the pro-stereotype and anti-stereotype speaker.

Moreover, as a more direct measure of attitudes toward gender stereotypes, we asked participants to indicate how problematic they found a set of descriptive and prescriptive stereotypical statements. We also added some new questions, asking participants how *true* they found the each of the descriptive (α = 0.86) and prescriptive (α = 0.80) stereotypes.

##### Exploratory items

We included five exploratory items to examine how participants perceive women who behave stereotypically. Examples include “women who use their femininity to get ahead are only putting themselves down in the long run (reverse coded),” and “women who use their femininity to get by are only making the best of difficult circumstances” (α = 0.62). We also included 2 items examining women's views on gender differentiation. Items were “the fact that women are different from men should be a point of pride,” and “women should try to disprove the idea that women are different from men” (reverse coded) (α = 0.52). Results for these measure are described in the Supplementary Materials.

#### Procedure

Data was collected through Qualtrics®. Participants accessed the study through the University of Groningen website. Participants first provided written informed consent and subsequently completed the measures in the same order as in Study 3. The new measure of gender differentiation and the exploratory items were completed at the end of the study. After completing all tasks, participants read a debriefing and were thanked.

#### Analytical strategy

As in Study 3, we assess the correspondence between findings of this study and those of Study 1 using multiple regression analysis, with identification with women and identification with feminists entered as predictors. When evaluating our hypotheses regarding the effects of the manipulation, we include the interaction between identification with women and identification with feminists as a third predictor in the multiple regression model. For the measure of “perceptions of stereotypes,” we also include the interaction term, and control for the perceived truth of the stereotype.

### Results

#### Identification with women and feminism

On average women identified strongly with their gender in-group (*M* = 5.41, *SD* = 1.04; 7-point scale), while identification with feminism was substantially lower (*M* = 3.35, *SD* = 1.51; 7-point scale). The correlation between identification with women and feminism was similar to that in Study 3 at *r* = 0.27 (*p* < 0.001). Table 6 shows the correlations between the different variables.

**Table 6:** Correlation table for Study 4.

		**ID with women**	**ID with feminists**	**Femininity**	**Modern sexism**	**Hostile sexism**	**Benevolent sexism**	**Descriptive problematic**	**Descriptive true**	**Prescriptive problematic**	**Prescriptive true**	**Anti speaker**
**ID with feminists**	Correlation	0.27	1									
	Significance	0.000										
**Femininity**	Correlation	0.57	0.22	1								
	Significance	0.000	0.002									
**Modern**	Correlation	0.14	0.50	0.09	1							
	Significance	0.052	0.000	0.203								
**Hostile**	Correlation	−0.08	−0.49	0.01	−0.38	1						
	Significance	0.293	0.000	0.939	0.000							
**Benevolent**	Correlation	−0.01	−0.30	0.10	−0.25	0.83	1					
	Significance	0.868	0.000	0.150	0.000	0.000						
**Descriptive problematic**	Correlation	−0.12	0.12	−0.09	0.15	−0.16	−0.20	1				
	Significance	0.089	0.088	0.222	0.036	0.023	0.005					
**Descriptive true**	Correlation	0.14	−0.14	0.12	−0.14	0.41	0.40	−0.30	1			
	Significance	0.044	0.045	0.105	0.046	0.000	0.000	0.000				
**Prescriptive problematic**	Correlation	−0.003	0.18	−0.04	0.35	−0.27	−0.27	0.29	−0.22	1		
	Significance	0.969	0.013	0.627	0.000	0.000	0.000	0.000	0.002			
**Prescriptive true**	Correlation	−0.04	−0.13	0.03	−0.24	0.42	0.48	−0.09	0.28	−0.51	1	
	Significance	0.587	0.078	0.687	0.001	0.000	0.000	0.203	0.000	0.000		
**Anti speaker**	Correlation	0.21	0.16	0.11	0.14	−0.1	0.01	−0.02	−0.02	−0.06	0.02	1
	Significance	0.003	0.023	0.139	0.049	0.168	0.855	0.834	0.747	0.392	0.754	
**Pro speaker**	Correlation	0.2	0.25	0.12	0.24	−0.17	−0.11	0.04	−0.04	0.23	−0.21	−0.05
	Significance	0.003	0.000	0.083	0.001	0.018	0.135	0.591	0.623	0.001	0.003	0.518

#### Correspondence to previous studies

As in previous studies, identification with women predicted attitudes toward group characteristics, and identification with feminists predicted attitudes toward the social position of the group. The statistical information for these findings is presented in Table 7. Higher identification with women led to higher perceptions of femininity. Those who identified strongly with feminism perceived more modern sexism in society, endorsed less hostile sexism, and less benevolent sexism.

**Table 7:** Attitudes predicted by identification with women, and identification with feminists in Study 4.

**Dependent**	**Predictor**	***B***	***SE***	***t-value***	***p-value***	**$R_{\text{change}}^{2}$**
Femininity	Identification with women	0.65	0.08	*t* = 8.59	*p* < 0.001	0.27
	Identification with feminists	0.05	0.05	*t < 1*	*p* = 0.308	0.003
Modern sexism	Identification with women	0.003	0.05	*t* < 1	*p* = 0.954	0.00001
	Identification with feminists	0.26	0.03	*t* = 7.69	*p* < 0.001	0.23
Hostile sexism	Identification with women	0.05	0.06	*t* < 1	*p* = 0.350	0.003
	Identification with feminists	−0.29	0.04	*t* = −7.71	*p* < 0.001	0.24
Benevolent sexism	Identification with women	0.07	0.07	*t* = 1.06	*p* = 0.291	0.005
	Identification with feminists	−0.20	0.04	*t* = −4.52	*p* < 0.001	0.10

#### Hypothesis test

##### Effects of the manipulation

As in Study 3, the manipulation of a conversation between a pro-stereotype and anti-stereotype speaker produced few theoretically interesting effects. Higher identification with women [*B* = 0.13, *SE* = 0.04, *t*_(195)_ = 3.22, *p* = 0.001, $R_{\text{change}}^{2}$ = 0.05] and feminists [*B* = 0.09, *SE* = 0.03, *t*_(195)_ = 3.40, *p* < 0.001, $R_{\text{change}}^{2}$ = 0.05] led to more positive ratings being given, regardless of the arguments put forward by the speakers. Unlike in Study 3, identification with women did not predict preference for either speaker (and neither did identification with feminists or the interaction, all *t*s < 1). In sum, our hypotheses regarding the manipulation were not supported.

##### Perceptions of stereotypes

**Figure 2:**
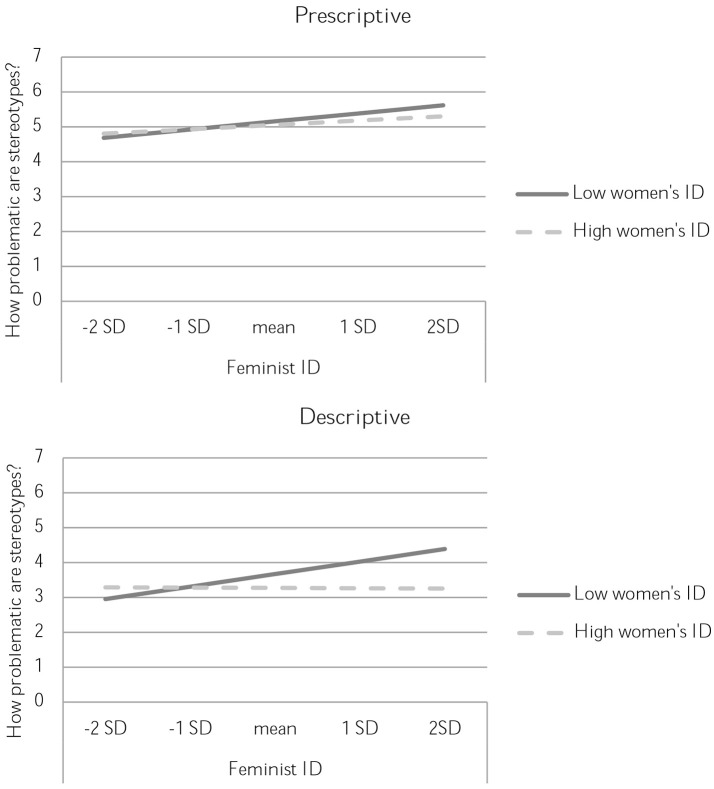
Perceptions of the problematic nature of stereotypes in Study 4, separated by valence of the stereotype and prescriptive **(top)** vs. descriptive **(bottom)** nature. High and low women's identification are plotted at ±1 standard deviation from the mean.

For prescriptive stereotypes, those who perceived the stereotypes as more true also perceived them as less problematic [*B* = −0.67, *SE* = 0.09, *t*_(195)_ = −7.95, *p* < 0.001, $R_{\text{change}}^{2}$ = 0.24]. Moreover, women who were strongly identified with feminists found prescriptive stereotypes more problematic than those who were not so strongly identified with feminists [*B* = 0.10, *SE* = 0.05, *t*_(195)_ = 2.09, *p* = 0.038, $R_{\text{change}}^{2}$ = 0.02]. Unlike in Study 3, there were no effects of identification with feminists on attitudes toward prescriptive stereotypes [*t*_(195)_ = −1.02, *p* = 0.309]. These findings are depicted in the top panel of Figure 2.

Descriptive stereotypes, too, were rated as less problematic by those who perceived them as more true [*B* = −0.32, *SE* = 0.08, *t*_(195)_ = −4.02, *p* < 0.001, $R_{\text{change}}^{2}$ = 0.07]. As in Study 3, the interaction between identification with women and identification with feminists predicted how problematic people found these stereotypes [*B* = −0.12, *SE* = 0.04, *t*_(195)_ = −2.60, *p* = 0.010, $R_{\text{change}}^{2}$ = 0.031]. Decomposition of the interaction showed that, at lower levels of identification with women, identification with feminists is an increasingly strong predictor of critical attitudes toward stereotypes. Put differently, the effect of identification with feminists is stronger when identification with women is lower [*B* = 0.23, *SE* = 0.08, *t*_(195)_ = 3.03, *p* = 0.003, $R_{\text{change}}^{2}$ = 0.042], as compared to higher (*t* < 1). This effect is depicted in the bottom panel of Figure 2. The alternative breakdown of the interaction revealed another significant simple slope: identification with women has a dampening effect on critical attitudes to gender stereotypes when identification with feminists is high [*B* = −0.33, *SE* = 0.11, *t*_(195)_ = −3.02, *p* < 0.003, $R_{\text{change}}^{2}$ = 0.04], but not when identification with feminists is low (*t* < 1). In Figure 2 bottom, this effect is evident from the fact that the lines representing lower vs. higher identification with women diverge more strongly at higher levels of identification with feminists.

In sum, results for the descriptive stereotypes confirmed our hypothesis, and replicated the interaction demonstrated in Study 3. Importantly, these patterns appear while controlling for the perceived truth of the stereotype.

### Discussion

Overall, the results of Study 4 correspond largely to those of Studies 1–3. As in Studies 1 and 2, identification with feminists reflected attitudes toward group relations, while identification with women reflected attitudes toward group characteristics. Importantly, the interaction from Study 3 was replicated, which showed that women who are more strongly identified with feminists are more critical of gender stereotypes, and this effect of identification with feminists is stronger when identification with women is *lower*. These findings regarding the interaction are in line with other recent work from our lab, which has examined responses to *implicit* gender stereotypes (van Breen et al., in preparation). Results in that line of studies show that a combination of stronger identification with feminists and *lower* identification with women as a group leads women to *resist* exposure to implicit gender stereotypes, for instance through persistence in counter-stereotypical performance domains. However, as in Study 3, the interaction effect was small. Therefore, in section Pooled Analysis, we further explore the reliability of the pattern. Study 4 also revealed some novel findings: perceptions of the problematic nature of gender stereotypes could not be explained by differences in the perceived truth of gender stereotypes.

Although those who are highly identified with feminists, *but not* with women (“distinctive feminists”) are most conspicuous in the results, theoretically speaking women who are highly identified on both dimensions (the “dual identifiers”) are also interesting. These women are feminists, but do not necessarily object to descriptive gender stereotypes. This finding may be due to the fact that stereotypes can provide differentiation from the out-group (i.e., men, see Brewer, 1991; Mlicki and Ellemers, 1996) which might be a desirable feature for those who are strongly identified with the group.

As in Study 3, the manipulation did not produce the expected effects in this study. Overall, participants agreed more with the arguments of the pro-stereotype speaker than the arguments of the anti-stereotype speaker. It may be the case that the anti-stereotype speaker was perceived as “too radical.” The anti-stereotype arguments were phrased quite prohibitively, such as “women should not behave stereotypically, as it reinforces the disadvantage women face.” Participants may have disliked this, and therefore favored the pro-stereotype speaker. An additional limitation of the manipulation was that both speakers expressed disapproval of women's low status position, and as such both speakers could be said to be feminists. Indeed, there is some evidence that lower identification with feminists was associated with lower agreement with the speakers overall (see Supplementary Materials). The disapproval of the low status position of women was kept constant, rather than varied, because the measure was designed to focus on perceptions of *stereotypes* as harmful or not. If we had also varied speakers' views on women's disadvantage, the conversation would have become very complex. Already there was some evidence that participants found it difficult to remember details of the conversation, and as such we considered it undesirable to further complicate the manipulation.

### Pooled analysis

Both Study 3 and Study 4 showed evidence that the interaction between identification with women and identification with feminists affects attitudes toward descriptive gender stereotypes. However, given the small size of the effect, we considered it worthwhile to assess this interaction in a *post-hoc* analysis with more power. As Study 3 and Study 4 had the same design, we can use Integrative Data Analysis (IDA; Curran and Hussong, 2009) to maximize power and evaluate the underlying pattern of the interaction. In this analysis, we pool the data from Studies 3 and 4 to assess whether, as in the individual studies, there is evidence that identification with women and identification with feminists interact to predict perceptions of descriptive gender stereotypes in the larger sample. Given a power of 1 − β = 0.80 and α = 0.05, the pooled sample (*N* = 380) can detect effect sizes of $R_{\text{change}}^{2}\approx$ 0.02 and above.

The pooled sample was analyzed with multiple regression analysis, in which, as before, identification with women, identification with feminists, and their interaction are entered as continuous predictors. Additionally, we added a dummy variable reflecting the Study from which each data point was derived, to control for the influence of the different samples.

Results showed that perceptions of descriptive stereotypes were affected by the interaction between identification with feminists and identification with women [*B* = −0.12, *SE* = 0.04, *t*_(379)_ = −3.18, *p* = 0.002, $R_{\text{change}}^{2}$ = 0.023]. Breakdown of the interaction showed the same patterns as those described above. Firstly, at lower levels of identification with women, identification with feminists is an increasingly strong predictor of critical attitudes toward stereotypes: the effect of identification with feminists is stronger when identification with women is low [*B* = 0.36, *SE* = 0.06, *t*_(379)_ = 6.11, *p* < 0.001, $R_{\text{change}}^{2}$ = 0.09], compared to when identification with women is high [*B* = 0.12, *SE* = 0.05, *t*_(379)_ = 2.43, *p* = 0.016, $R_{\text{change}}^{2}$ = 0.01]. The alternative breakdown of the interaction revealed another significant simple slope: identification with women has a dampening effect on critical attitudes to gender stereotypes when identification with feminists is high [*B* = −0.46, *SE* = 0.09, *t*_(379)_ = −5.09, *p* < 0.001, $R_{\text{change}}^{2}$ = 0.06], but not when identification with feminists is low (*t* < 1).

These findings confirm that the interaction found in Studies 3 and 4 is reliable when assessed in the pooled data set. Taken together, the different patterns that make up the interaction show that critical attitudes toward descriptive gender stereotypes are strongest amongst women who are strongly identified with feminists, but less so with the broader group of women.

## General discussion

The studies presented here provide insight into how identification with women and feminists predict different attitudes toward gender as a social category. We now review the results of the studies in the light of the multiple identities approach, and evaluate its utility in predicting attitudes toward gender issues.

### The multiple identities approach

The multiple identities approach proposes that attitudes toward gender as a social category are determined by two distinct dimensions of gender identity: identification with women, reflecting attitudes toward the characteristics associated with the group, and identification with feminists, reflecting attitudes toward the social position of the group. This central prediction of the model is confirmed across the four studies reported here, in student samples as well as a community sample. That is, the studies confirm that identification with women and identification with feminists represent distinguishable aspects of gender identity, and as such, that gender identity is not unitary (Condor, 1986; Henderson-King and Stewart, 1994; Becker and Wagner, 2009). Moreover, results show that identification with women is related to attitudes toward group characteristics, such as femininity and self-stereotyping. These “group characteristics” need not be thought of as essentialist traits, but rather as part of a culturally shared understanding of the social category of “women” (Devine, 1989; Rudman and Glick, 2008). Identification with feminists, by contrast, is related to attitudes toward the group's social position, such as support for collective action and perceptions of sexism.

#### Combining identification with women and feminists

If we consider gender identity in the light of the multiple identities approach, this gives rise to the question of how the dimensions may be combined. The multiple identities approach suggests that, when a certain issue has a bearing on both group characteristics and the group's social position, attitudes toward such an issue will be affected by both identification with women and identification with feminists. Indeed, studies 2–4 showed that issues such as support for radical collective action and perceptions of gender stereotypes are affected by both identification with women *and* identification with feminists, manifested as additive or interactive effects. The finding that particular combinations of identification with women and feminists lead to differences in attitudes toward gender issues is not only in line with the multiple identities approach, but also corresponds to other recent work from our lab (van Breen et al., in preparation), as well as the findings of Leicht et al. (2017, this issue).

The combinations of different gender identities can be thought of in terms of different conceptual groups or “prototypical types” of gender identifiers. In fact, several theorists have found it helpful to discuss the possibility of gender identity “subgroups” to address the question how different aspects of gender identity relate to one another (Condor, 1986; Gurin and Markus, 1989; Becker and Wagner, 2009). In our approach, the first possible combination includes those whose identification with both women and feminists is relatively low (“low identifiers”). Low identifiers navigate gender group membership by giving priority to social identities *outside* the gender context, as they dislike being viewed in terms of gender (Barreto et al., 2010). Secondly, there are those who identify strongly with women but not feminists (“traditional women”). Traditional women value typically female gender roles (Condor, 1986), but they disavow feminist concerns about the social position of women. There are two feminist subgroups: those who are highly identified with feminists *and* women (“dual identifiers”; see Leicht et al., 2017 in this issue), and those who are highly identified with feminism, *but not* women (whom we have called “distinctive feminists”). Dual identifiers can be described as preferring integrative identity management strategies that unite their commitment to women as a group with their commitment to feminism. For instance, they may be willing to take on leadership positions (Leicht et al., 2017; this issue), but prefer more feminine styles when they do so (Olsson and Walker, 2004). Distinctive feminists, on the other hand, navigate gender group membership by giving priority to feminist issues over their identification with women. For instance, they may disavow feminine beauty ideals because they perceive them as contributing to women's objectification (Murnen and Smolak, 2009). It is important to note that even though “distinctive feminists” do not identify highly with women, this does not mean that they are “anti-women” (see Becker et al., 2011; Cichocka et al., 2013). Rather, they disavow the (current) *social construction* of the group.

Importantly, this taxonomy does not represent fixed or absolute categories, but rather a way of conceptualizing different approaches to integrating the identities. Indeed, we see gender identity as dynamic and context-dependent. Given that the social construction of identity plays a large part in our approach, arguably the most important contextual factor is the nature of the social construction. Different cultures may construct gender differently, and this may in turn affect attitudes to specific gender issues. Additionally, an individual's commitment to the different identities may develop over time, for instance through personal experience. Likewise, research on social influence has shown that making salient an intergroup context can shift individuals' attitudes toward those of more radical minorities within the in-group (David and Turner, 1999). As we used cross-sectional data we did not examine this dynamic component of multiple identities in the current study, but we believe this is a fruitful area for future research.

In sum, the different combinations of high vs. low identification with women and feminists can be thought of as reflecting different strategies for managing multiple gender identities. Some women prioritize one dimension over the other (traditional women; distinctive feminists) while others seek to integrate them (dual identifiers).

### Advantages of the multiple identities approach

The multiple identities approach has several advantages that are worth highlighting. Firstly, the fact that identification with women and identification with feminists represent separable components of gender identity allows for different kinds of identity content, which is crucial when attempting to model something as diverse as attitudes toward gender group membership. One consequence of this is that identification with feminists and femininity are not mutually exclusive: a woman may embrace both femininity and feminism. As noted above, this issue is also reflected in feminist discourse (Gilligan, 1977; Butler, 2002). A further consequence of the two independent dimensions is that some women are highly identified with women as a group, but do not hold politicized identities. Indeed, our findings on collective action confirm that high identification with women does not automatically increase politicized attitudes (Henderson-King and Stewart, 1994).

As identification with feminists can function independently of identification with women, identification with feminists can also be part of men's gender identity (e.g., Digby, 2013). Preliminary results of applying the multiple identities approach to men's gender identity^6^ show that, as amongst women, identification with men as a group correlates with perceived masculinity and self-stereotyping, while stronger identification with feminists increases perceived prevalence of sexism. However, the relationship between the identities is somewhat different amongst men: for men the factors are negatively correlated; those who identified more strongly with men, and felt more masculine, were less likely to identify with feminism (see also Burn et al., 2000; Lemaster et al., 2015). In sum, the possibility of applying the multiple identities approach to men's gender identity allows us to assess how men's attitudes toward gender group membership differs from women's, as well as where similarities lie. Though further work is needed on this front, we consider this a strength of the model.

The distinction between group characteristics and the group's social position may also play an important role in how people think of identities outside the gender context, such as ethnic group membership. For instance, we can think of the multiculturalist approach to ethnic diversity as appreciating group differences while also addressing political disadvantage (Verkuyten and Brug, 2004), suggesting that, as the multiple identities approach argues, both attitudes to group characteristics and perceptions of the group's social position play a role in how social group membership is constructed.

A further methodological strength of this approach is its concise measure of identification, using eight items in total to measure identification with women and identification with feminists. The two identification variables were measured with the same items, apart from the fact that the word “women” was replaced by “feminists.” Thus, these gender identity dimensions are shown to be independent, even when the measures are very similar. Therefore, the lack of correlation between identification with women and identification with feminists is a conservative test of the independence of the dimensions.

A limitation of the current study is its correlational nature, preventing inferences about causal direction. For instance, the relationship between identification with feminists and perceived sexism might arise because identification with feminists leads to increased sensitivity to sexism (Major et al., 2003) or, conversely, increased exposure to sexism might lead to increased identification with feminism (Henderson-King and Stewart, 1994). In fact, it is likely that both these processes play a part in identity development. A further limitation is the reliance on student samples in Studies 1, 2, and 4. Students are likely to hold more progressive attitudes than the general population, and therefore it may be more possible for the same person to identify with both women and feminists amongst students than it is in the general population. That is, it is possible that in other populations there would be a negative correlation between the two identities. However, in Study 3, which used a community sample, there was no evidence for such a negative correlation. Findings from Study 3 instead tended toward a positive correlation between identification with women and identification with feminists. Nevertheless, the reliance on student samples is an additional limitation of this study.

### Conclusions

This study develops the multiple identities approach to gender identity, in which identification with women and identification with feminists are orthogonal components of gender identity, which together predict attitudes toward gender group membership. Identification with women predicts attitudes toward group characteristics, such as perceived femininity and self-stereotyping, while identification with feminists predicts attitudes toward the group's social position, such as sexism and disadvantage for women. Different combinations of identification with women and feminists give rise to four conceptual identity profiles: low identifiers, traditional women, distinctive feminists, and dual identifiers. Importantly, the multiple identity approach helps to explain differences in gender attitudes, notably that: (1) Strong identification with feminists does not preclude a sense of being feminine; (2) Strong identification with women as a group does not automatically increase politicized attitudes; and (3) Critical attitudes toward gender stereotypes are most pronounced amongst feminists who are less strongly identified with women. Taken together, findings from these studies suggest that considering identification with women and identification with feminists as multiple identities can provide valuable new insights into attitudes toward gender group membership.

## Ethics statement

The studies reported in this article conform to APA ethical guidelines, and have been approved by the relevant local ethical committees. The studies conducted at the University of Groningen have been approved by the Ethical Review Board of the University, which bases its approval on guidelines from the professional code of the NIP, and the Personal Data Protection Act. More information can be obtained from http://www.rug.nl/research/heymans-institute/organization/ecp/ or the first author.

## Author contributions

All authors contributed to study conception and design. Acquisition and analysis of data was conducted by JB and SdL. JB drafted the manuscript, RS, TK, and SdL gave feedback for its revision.

### Conflict of interest statement

The authors declare that the research was conducted in the absence of any commercial or financial relationships that could be construed as a potential conflict of interest.

## Footnotes

^1^Adding the interaction term of identification with women and feminists did not substantially change these results.

^2^Adding the interaction term of identification with women and feminists did not substantially change these results.

^3^These results are not substantially changed when controlling for the manipulated factors.

^4^We use multiple regression analysis here to maintain consistency across measures and across studies. However, we also repeated this analysis using repeated measures ANCOVA. In the RM ANCOVA, the identification variables and their interaction were entered as covariates and the different Speakers as the within-participants factor. Results of the RM ANCOVA were equivalent to outcome of the multiple regression analyses (described below), as these two analytical techniques are based on the same procedure (Edwards, 1985; Judd et al., 2011).

^5^Aside from the differences between the speakers, there were main effects of both identification variables on evaluations of the speakers (regardless of the arguments they put forward): higher identification with feminists (*B* = 0.11, *SE* = 0.03, *t*_(183)_ = 3.53, *p* = 0.001, $R_{\text{change}}^{2}$ = 0.05), and higher identification with women (*B* = 0.23, *SE* = 0.05, *t*_(183)_ = 4.24, *p* < 0.001, $R_{change}^{2}$ = 0.09) led to more positive ratings. Finally, there was an interaction between identification with women and identification with feminists [*B* = −0.07, *SE* = 0.03, *t*_(183)_ = −2.12, *p* = 0.035, $R_{\text{change}}^{2}\;=$ 0.02], such that women who were low identifiers on both dimensions gave especially low ratings. As these effects are independent of the arguments given by the speakers, scope for interpretation is limited.

^6^Further details of this work can be obtained from the first author.
