# Supplementary material for: A Multiple Identity Approach to Gender: Identification with Women, Identification with Feminists, and Their Interaction
Source: Front Psychol. 2017 Jun 30;8:1019. doi: 10.3389/fpsyg.2017.01019 (PMC5492047; doi:10.3389/fpsyg.2017.01019)
Supplement: Supplementary file 1 [file DataSheet2.pdf]

Supplementary Materials

These supplementary materials accompany the manuscript "A multiple identity approach to gender: Identification with women, Identification with feminists, and their interaction" (van Breen, Spears, Kuppens, & de Lemus; doi: 10.3389/fpsyg.2017.01019).

Study 1

In this section readers can find a description of some additional measures included in Study 1 that are not described in the main text of the article.

Method.

Inclusion of the self in other scale. This scale requires participants to select one from a series of 7 pictures (Schubert & Otten, 2002). Each picture shows a circle labelled "self" and a larger circle labelled with the group name. Consecutive pictures show decreased distance between the self and the group. Thus, while the first picture shows considerable separation of the self and the group, in the final picture the self is completely within the group circle. Two versions of this scale were used, one asking about the distance between self and women as a group and the other asking about the distance between women and men.

Gender Role Preferences. The 8 items of the gender role preference scale (Becker & Wagner, 2009) examine participants' gender role preferences (α=0.80). This scale was included to compare results for our measure of identification with feminists to results for this scale used by Becker and Wagner (2009).

Results.

Inclusion of the self in women as a group. The extent to which participants perceived overlap between themselves and women as a group, was predicted by both identification with women (B=0.18, SE=0.04, $t(1,85)$=4.47, p< 0.001), and identification with

feminists (B=0.05, SE=0.02, $t(1,85)=2.17$, $p=0.033$). The item asking about the overlap between women as a group and men as a group was not predicted by either of the identification variables (ts<1.77, ps>0.81).

Gender role preference. Scores on the Gender Role Preference scale were not predicted by identification with women or identification with feminists (ts<1).

Study 2

In this section readers can find a description of several additional measures included in Study 2 that are not described in the main text of the article.

Method.

As data for this study were collected as part of a larger experiment, there were several dependent variables that are not of central interest to the current study. These measures are described here, in the order in which they were administered.

Support for Feminist Goals. Attitudes to the feminist movement were measured with the global goals of feminism scale (Morgan, 1996), consisting of 9 items such as "Women should be considered as seriously as men as candidates for the Presidency of Spain" ($\alpha=0.56$), rated on a scale of 1 to 7.

Affirmative action attitudes. Attitudes towards affirmative action aimed at improving the position of women were measured by 3 items (from Tougas et al, 1999; e.g., "If there are no affirmative action programs helping women in employment, they will continue to be unfairly treated"; $\alpha=0.63$), rated on a scale of 1 to 7.

Emotions. Participants rated the extent to which the manipulation led them to feel a range of positive (N=3, $\alpha=0.58$), and negative (N=5, $\alpha=0.74$) emotions.

Study 3

In this section readers can find a description of additional exploratory measures included in Study 3 that are not described in the main text of the article.

Method.

Manipulation text. Below readers can find the text of the within-participants manipulation. The order of the arguments was counterbalanced, so that half of the participants read the arguments in the order pro-against-pro-against, and some participants read the arguments in the order against-pro-against-pro.

"On the next page you will read an extract from a discussion between two women, Sara and Ellie, who are discussing why women continue to be under-represented in leadership positions. Please read their arguments carefully. After reading through the conversation, you will be asked to give your opinion on both of the speakers.

Sara: *I think that the reason that women are underrepresented in leadership is that women are stereotyped as having certain qualities, like being sweet and caring. Those stereotypes are used to suggest that women are unsuitable for leadership or other influential positions in business. I think women can make very good leaders, but stereotypes hold them back. So, I think that stereotypes are the reason that we see so few women in leadership positions.*

Ellie: *I would say that stereotypes are not necessarily problematic, rather, the problem is that the qualities women have are undervalued in our society. For instance, the fact that women are more sensitive and less aggressive than men could make women very good leaders, but society as a whole has not realised that yet. Basically, I think women are underrepresented in leadership because feminine qualities are undervalued.*

Sara: *I think it is not just a matter of getting people to value women's qualities more. IN fact I would say it is harmful to talk about "women's qualities" versus "male qualities",*

because this will be used as justification for treating men and women differently. This ultimately reinforces the disadvantage that women in leadership positions are already exposed to.

Ellie*: I don't think the distinction between "women's qualities" versus "male qualities" is such a problem. We need all types of people with different qualities, and if women are comfortable with more feminine styles then that is no problem. In fact, I think that taking a feminine approach can bring many advantages, like improved communication. What I think is a problem is when people then start to assume that women can't do other things, like lead a negotiation, just because they take a stereotypically feminine approach.*

Please click through to the next page to indicate the impression the speakers Sara and Ellie made on you."

Hiring task. In the hiring task, the instructions asked participants to imagine that a new female leader had been hired at a company they worked for, and that this woman would become their new department boss. Participants were then asked to evaluate a series of traits in terms of how important they would be for their new female leader to have. Items focused on warmth (α=0.77), competence (α=0.65), and morality (α=0.61). This measure was designed to examine perceptions of stereotypes indirectly; more endorsement of stereotypes would lead to a preference for more stereotypical attributes (i.e. warmth).

Regulatory focus. Research has shown that individuals under promotion focus commit to collective action when it is likely that the objectives will be achieved. Individuals under prevention focus, however, saw collective action for social change as a moral obligation, and were less affected by the likelihood of success (Zaal, Van Laar, Ståhl, Ellemers, & Derks, 2012). Extending this reasoning to the gender context, those who are more strongly identified with women and feminists may adopt a promotion focus when considering gender issues: they understand the disadvantaged social position of women, but are quite

satisfied on a personal level, and therefore would only "risk" engaging in collective action when it is likely that the objectives are reached. Conversely, those who are strongly identified with feminists *but not* women, may adopt a prevention focus: they see striving for social change on gender issues as a moral obligation. This option was explored as an alternative to our central hypothesis. We measured regulatory focus, both dispositional, and in the context of gender issues. Dispositional regulatory focus was assessed with the Regulatory Focus Proverb Questionnaire (N=12, α=0.71, Van Stekelenburg, 2006). Participants rate the extent to which proverbs with promotion (N=6) and prevention (N=6) foci reflect the approach they take to life. Examples include "better be safe than sorry" (prevention), and "nothing ventured nothing gained" (promotion).

Alongside this dispositional measure of regulatory focus, we developed some items that reflect situational regulatory focus of statements, specific to the gender context. Six items (α=0.80) examined endorsement of feminist principles when they were framed as having either prevention goals (preventing sexism) or promotion goals (promoting gender equality). Another 5 items (α=0.97) represented the objectives of (moderate) collective action as either preventive or promotion-focused (i.e. "drawing attention to the disadvantage faced by women" vs. "showing support for women's rights").

Attitude strength. We wished to exclude the alternative possibility that the interaction between identification with women and feminists affects attitude strength rather than content. Such an explanation would be counter to multiple identities approach, which distinguishes the subgroups based on content, rather than strength of identification. The measure of attitude strength was composed of 8 items (α=0.88, items adapted from Vonofakou, Hewstone, & Voci, 2007), such as "How often do you think about gender and its meaning?".

Results.

Hiring task. In the hiring task, participants were asked to rate the importance of several traits representing warmth, competence and morality, in terms of how important they would be for a new female leader in their company to have. There was a significant 3-way interaction between identification with women, identification with feminists, and dimension, $F(1, 184)=4.29$, $p=0.040$, such that the differences between the dimensions are amplified as scores on the identification variables go up. Those women who are not strongly identified with either women or feminists do not differentiate between dimensions in their importance ratings ($F<1$), while those who are highly identified with both women and feminists rate all dimensions differently ($F(1, 184)=13.89$, $p<0.001$), finding morality the most important, and competence the least important.

In sum, though there was evidence for an interaction between identification with women, identification with feminists and the trait dimension, these patterns did not support our hypothesis that preference for stereotypical (warmth) traits would vary as a result of identification with women and feminists. Instead, findings suggest that as identification goes up, opinions on the traits of female leaders become more pronounced, such that high/high identifiers appear to have stronger preferences than low/low identifiers.

Regulatory focus. The first component of the regulatory focus measure assessed dispositional regulatory focus through the RFPQ (Van Stekelenburg, 2006), to examine general preference for prevention and promotion focus. Results showed that, in general, participants reported more agreement with promotion focused items than prevention focused items ($F(1, 184)=6.36$, $p=0.013$). Additionally, there was a main effect of identification with women ($F(1,184)= 4.46$, $p=0.036$), such that higher women's identifiers endorsed the statements more, regardless of their focus.

Endorsement of feminism was affected by a main effect of identification with feminists, ($F(1,184)= 57.31$, $p<0.001$, such that higher identification with feminists lead to

more endorsement of the aims of feminism, regardless of their prevention or promotion focus (Fs<1.42, ps>0.236). Additionally, there was a main effect of focus, such that participants in general perceived feminism as having a preventive focus (i.e. preventing women's disadvantage, rather than supporting women's rights), $F(1, 184)=18.21, p<0.001$.

Endorsement of gender-related collective action also showed a main effect of identification with feminists, $F(1, 184)=79.97, p<0.001$, such that higher identification with feminists increased endorsement of collective action. Again, there was a main effect of focus, $F(1, 184)=5.13, p=0.025$, such that participants endorsed more collective action when it focused on preventing women's disadvantage.

There was no evidence for an interaction between identification with women, identification with feminists and regulatory focus on any of the three measures ($Fs<1$).

Attitude Strength. Attitude strength was associated with identification with feminists, $t(184)=15.41, p<0.001$, such that higher identification with feminists predicted stronger attitudes on gender issues. Crucially, the interaction between identification with feminists and identification with women did not reach significance ($t<1$). Thus, there is no evidence that the interaction between identification with women and identification with feminists produces differences in the strength of attitudes.

Study 4

In this section readers can find a description of additional exploratory measures included in Study 4 that are not described in the main text of the article.

Method.

Hiring task. In the hiring task, the instructions asked participants to imagine that a new female leader had been hired at a company they worked for, and that this woman would become their new department boss. Participants were then asked to evaluate a series of traits in terms of how important they would be for their new female leader to have. Items focused

on warmth (α=0.77), competence (α=0.76), and morality (α=0.68). This measure was designed to examine perceptions of stereotypes indirectly; more endorsement of stereotypes would lead to a preference for more stereotypical attributes.

Regulatory focus. The previous study measured regulatory focus generally, as well as regulatory focus when considering gender issues. In this Study, we focus on the measures that framed the aims of feminism in either promotion or prevention terms. In the previous study, those who were more strongly identified with feminists tended to agree with all statements, regardless of the regulatory focus it reflected. Therefore, in the current study these items were presented as forced choice: participants were asked to select the option that reflected their opinions most closely, with on one end of the scale a preventively framed option (e.g. the aim of feminism is to prevent sexism) and at the other end of the scale a promotion-focused option (e.g. the aim of feminism is to promote gender equality). Using the statements as opposite scale anchors halved the number of items (N=3, α=0.60).

Attitude strength. This measure examined the alternative possibility that identification with women and feminists affects attitude strength rather than content. Such an explanation would be counter to the TGIF model, which distinguishes the subgroups based on content, rather than strength of identification. The measure of attitude strength was composed of 8 items (α=0.81, items adapted from Vonofakou et al., 2007), such as "How often do you think about gender and its meaning?"

Gender differentiation. We also included 2 items examining women's views on gender differentiation. Items were "the fact that women are different from men should be a point of pride", and "women should try to disprove the idea that women are different from men" (reverse coded) (α=0.52).

Results.

Hiring task. In the hiring task, participants were asked to rate the importance of several competence and warmth-related traits for a hypothetical female leader. Results showed that there was an interaction between feminist and identification with women, $F(1,195)=4.79$, $p=0.030$, such that low identifiers placed less importance on a female leader having positive traits than other women did.

Positive traits also represented different dimensions, of warmth, competence and morality. There was a main effect of dimension, $F(1,195)=72.09$, $p<0.001$, such that participants overall placed greatest importance on morality, followed by warmth, followed by competence.

Regulatory Focus. There were no significant effects of feminist or identification with women on regulatory focus (Fs<1.038, $p>0.309$).

Attitude Strength. As in Study 3, attitude strength was positively predicted by identification with feminists, B=0.29, SE=0.03, $t(195)=8.98$, $p<0.001$, such that feminist identifiers reported stronger gender attitudes than non-feminist identifiers. The interaction between identification with feminists and identification with women did not reach significance ($t<1.43$, $p>0.156$).

Gender differentiation. Overall, identification with women predicted support for gender differentiation (B= 0.22, SE=0.09, $t(195)=2.42$, $p=0.016$). Moreover, there was a marginal interaction between identification with women and identification with feminists (B= 0.91, SE=0.06, $t(195)=1.66$, $p=0.098$), showing that the effect of feminist identification was somewhat stronger amongst lower women's identifiers. However, given the low reliability of the scale, interpretability of this finding is limited.

Exploratory items. We included exploratory items to examine judgments of stereotypical behavior by other women. Results showed no significant effects of the identification variables or their interaction on these items (ts<1.01, $p>0.271$).
